# Lectin staining and Western blot data showing differential sialylation of nutrient-deprived cancer cells to sialic acid supplementation

**DOI:** 10.1016/j.dib.2015.09.043

**Published:** 2015-10-09

**Authors:** Haitham A. Badr, Dina M.M. AlSadek, Mohit P. Mathew, Chen-Zhong Li, Leyla B. Djansugurova, Kevin J. Yarema, Hafiz Ahmed

**Affiliations:** a Department of Biochemistry, Faculty of Agriculture, Zagazig University, Zagazig 44511, Egypt; b Department of Histology and Cytology, Faculty of Veterinary Medicine, Zagazig University, Zagazig 44511, Egypt; c Department of Biomedical Engineering and Translational Tissue Engineering Center, The Johns Hopkins University, 400 North Broadway Street MD, Baltimore 21231, USA; d Department of Biomedical Engineering, Florida International University, 10555 West Flagler Street, Miami, FL 33174 USA; e Institute of General Genetics and Cytology, Al-Farabi Ave, 93, Almaty 050060, Kazakhstan; f Department of Biochemistry and Molecular Biology, University of Maryland School of Medicine and Institute of Marine and Environmental Technology, 701 East Pratt Street, Baltimore, MD 21202, USA

**Keywords:** Differential sialylation, Aberrant glycosylation, Sialic acid biosynthesis, Nutrient deprivation, Metabolic glycoengineering.

## Abstract

This report provides data that are specifically related to the differential sialylation of nutrient deprived breast cancer cells to sialic acid supplementation in support of the research article entitled, “Nutrient-deprived cancer cells preferentially use sialic acid to maintain cell surface glycosylation" [1]. Particularly, breast cancer cells, when supplemented with sialic acid under nutrient deprivation, display sialylated glycans at the cell surface, but non-malignant mammary cells show sialylated glycans intracellularly. The impact of sialic acid supplementation under nutrient deprivation was demonstrated by measuring levels of expression and sialylation of two markers, EGFR1 and MUC1. This Data in Brief article complements the main manuscript by providing detailed instructions and representative results for cell-level imaging and Western blot analyses of changes in sialylation during nutrient deprivation and sialic acid supplementation. These methods can be readily generalized for the study of many types of glycosylation and various glycoprotein markers through the appropriate selection of fluorescently-labeled lectins.

**Specifications Table**

**Table:** 

Subject area	*Biology*
More specific subject area	*Nutrient deprivation, cell surface glycosylation*
Type of data	*Cell images, Western blot*
How data was acquired	*Imaging cells on DV elite imaging system from Applied Precision (Applied Precision, USA).*
*Western blotting using chemiluminescent substrate (Pierce*™ *Fast Western Blot Kit and ECL Substrate from Thermo Fisher Scientific Inc.).*
Data format	*Filtered images for cell images, data for Western blot*
Experimental factors	*Cells were grown on the surface of a cover slip and the adherent cells were fixed with 70% ice-cold ethanol. After fluorescent-labeled lectin staining, cells were further treated with Ribonuclease A and the nuclei were counter stained with TO-PRO-3.*
*The protein expression and sialylation of EGFR and MUC1 was examined by Pierce ECL Western Blotting system.*
Experimental features	*Cells were stained with different FITC-labeled lectins and the nuclei were counter stained with TO-PRO-3. Images were captured on DV elite imaging system and merged using softWoRx DMS from Applied Precision (Applied Precision, USA).*
*Protein extracts of cells were immunoprecipitated and immunoprecipitated proteins were subjected to Western blotting.*
Data source location	*The Delta Vision Elite Imaging System Core Facility at Herbert Wertheim College of Medicine, Florida International University.*
*Immunoprecipitation and Western blotting were performed at Florida International University and Zagazig University.*
Data accessibility	*The data are provided in this article.*

**Value of the data**

• The supplementation with sialic acid (Neu5Ac) resulted in higher expression of Neu5Ac on cancer cells than normal cells. In cancer cells, the expression of Neu5Ac was notably on the cell surface, whereas in normal cells expression of Neu5Ac was intracellular.

## 1 Data

The data show that cancer cells under nutrient deprivation conditions use sialic acid to maintain cell surface glycosylation and surface glycan display.

### 1.1 Neu5Ac treatment of nutrient deprived cancer cells enhances sialylation at the cell surface

**Fig. 1:**
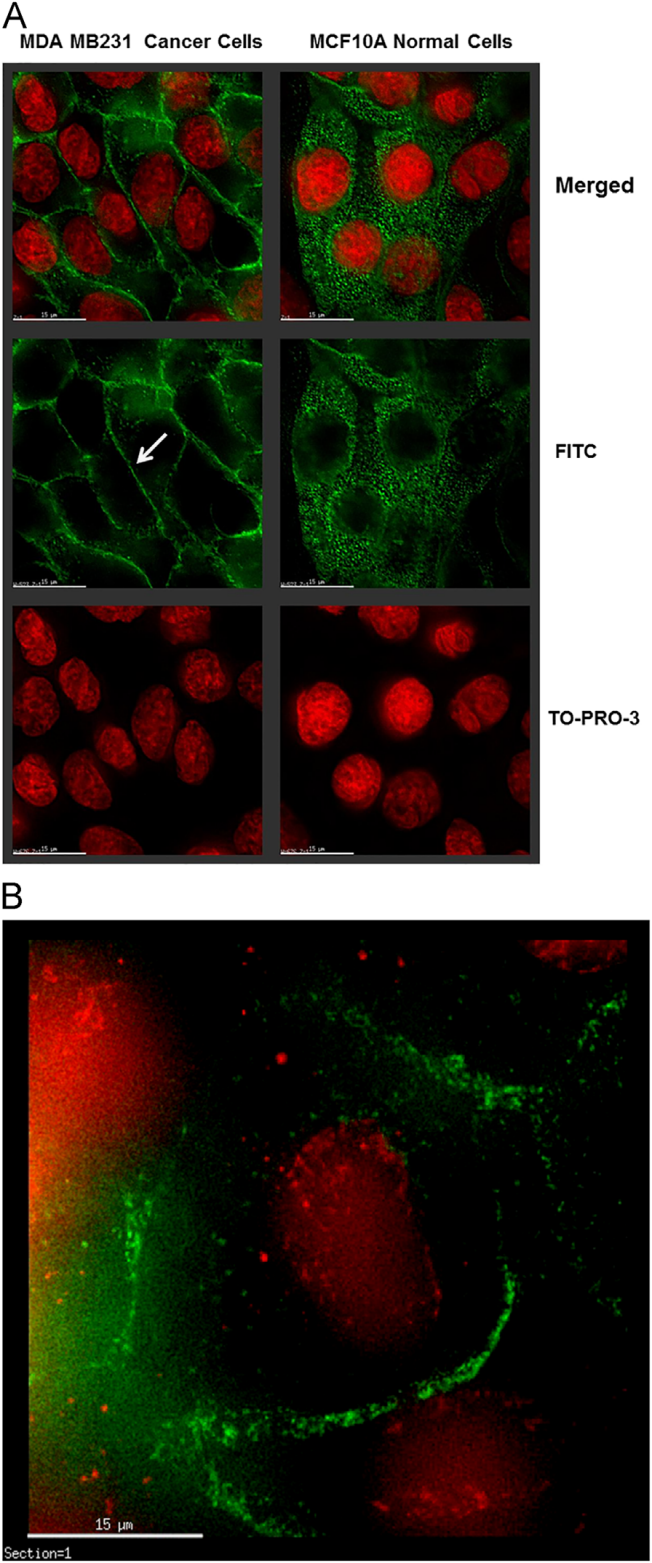
(A) MAL-I lectin staining followed by cell imaging**.** Cells were treated for 2 h in the presence of Neu5Ac (10 mM) under nutrient deprivation and stained for 30 min with FITC-labeled MAL-I lectin at concentration of 5 µg/mL (green fluorescence). Cells were further treated with 1 µg/ml RNase A and the nuclei were counter-stained with TO-PRO-3 (red fluorescence), Images are shown at 15 µm magnification. The white arrow represents MAL-I binding of cell surface glycans. © The Delta Vision Elite Imaging System Core Facility at Herbert Wertheim College of Medicine, Florida International University, Miami (File date: 13-12-2012). (B) Blow up of MAL-I staining of MDA MB231 cancer cell showing cell surface glycan sialylation (green region).

Supplementation of breast cancer cells (*T47D, MCF7, and MDA MB231) and normal mammary cells (MCF10A and HB4A)* to 10 mM sialic acid (Neu5Ac) under nutrient deprivation for 2 h (optimization of treatment condition described in the main article [1]) resulted enhanced sialylation as *quantitated* by binding with WGA (specific for Neu5Ac), SNA (specific for Neu5Ac-α2,6Gal), and MAL-I (specific for Neu5Ac-α2,3Gal) using flow cytometry of the permeabilized cells [1], [2]. Interestingly, the enhanced sialylation of the nutrient deprived cells upon sialic acid supplementation was more pronounced in the cancer cells (1.5-to-2.8-fold) compared to the normal cells (1.4-to-1.5-fold). The increased lectin binding to the Neu5Ac-supplemented, nutrient-deprived cells was corroborated by confocal microscopy. Consistent with the flow cytometry data, FITC-conjugated WGA, SNA, and MAL-I lectins showed enhanced fluorescence signal for both normal and cancer cells after sialic acid treatment [1]. Interestingly, MAL-I staining in the treated normal cells was distributed throughout the Golgi as demonstrated by co-localization with the Golgi marker GM130, but in the malignant cells this lectin was located almost exclusively on the cell surface [1] (see also DIB Fig. 1A and B). In contrast, control experiments where both normal and malignant cells treated with Neu5Ac (10 mM for 48 h) in complete medium showed only minimal enhancement (e.g., 6–28%) of lectin binding (Figure S3 in Ref. [1]).

### 1.2 Neu5Ac treatment of nutrient deprived cancer cells increases the expression and sialylation of EGFR and MUC1

**Fig. 2:**
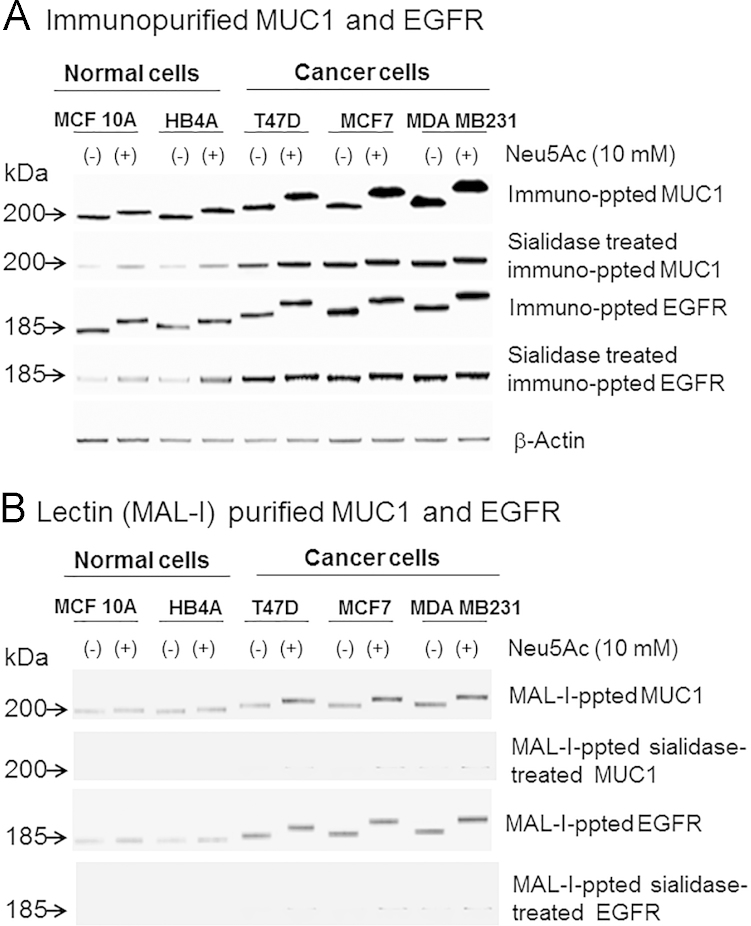
Examination of sialylation of MUC1 and EGFR on normal and malignant cells after sialic acid treatment under nutrient deprivation. (A) Equal amount (100 μg) of each cell extract was subjected to immuno-precipitation with anti-MUC1 and anti-EGFR antibodies and the precipitated proteins were subjected to Western blot and immuno-detection with the respective antibody. In parallel, equal amount of each crude protein extract was desialylated and similar precipitation was carried out. Please note, both MUC1 and EGFR purified from the Neu5Ac treated cancer cells (T47D, MCF7, and MDA MB231) moved slower on SDS-PAGE compared to the normal cells (MCF10A and HB4A), but this effect was reversed by sialidase treatment (see corresponding lower panel) suggesting that MUC1 and EGFR from the Neu5Ac treated cancer cells were more sialylated compared to those from the normal cells. (B) Equal amount of immuno-precipitated proteins (MUC1 and EGFR) as described above was subjected to MAL-I precipitation and detected on Pierce ECL Western Blotting as described in Materials and Methods. As expected, negligible or no bands were observed when immunopurified MUC1 and EGFR were first sialidase-treated to remove sialic acid and then precipitated with MAL-I (corresponding lower panel). As MAL-I binds α2→3-sialylated glycoproteins, no precipitation occurred with sialidase treated MUC1 and EGFR.

To explore the importance of sialic acid supplementation of nutrient-deprived cells on specific membrane glycoproteins, two cell surface receptors (EGFR and MUC1) were chosen because of their important roles in promoting tumor growth and metastasis. To determine whether EGFR and MUC1 were over-expressed, over-sialylated, or both, EGFR and MUC1 were immuno-precipitated and analyzed by Western blot analysis using commercial antibodies that recognized the un-glycosylated form of each protein. The anti-MUC1 antibody recognizes an epitope corresponding to the tandem repeat region of MUC1and detects only one band in MCF7 cell line (Product Data Sheet, Santa Cruz) as well as in other cell lines (Personal Communication, Santa Cruz Technical Service). Anti-EGFR antibody also detected only one band. Negative control experiment for each antibody did not result in any band (data not shown). As shown in DIB Fig. 2A, anti-MUC1 and anti-EGFR antibodies immuno-precipitated increased amounts of MUC1 and EGFR from malignant cell lysates compared to the normal cells. The higher levels of MUC1 and EGFR immunopurified by antibodies that recognize protein epitopes of these glycoproteins was consistent with qRT-PCR results where mRNA levels of these two proteins in nutrient-deprived cells supplemented with sialic acid were compared to the untreated cells (Figure S4 in Ref. [1]). Moreover, MUC1 and EGFR purified from the Neu5Ac treated malignant cells were more heavily sialylated (based on slower migration observed during SDS-PAGE) compared to those from the untreated cells (both normal and malignant) as well as treated normal cells, an effect that was reversed by sialidase treatment (DIB Fig. 2A). To further corroborate the increased sialylation of these cancer markers, equal amounts of immunoprecipitated MUC1 and EGFR were precipitated with MAL-I and subjected to Western blot analysis followed by immunodetection that again showed that the malignant cells migrated slower during SDS-PAGE consistent with increased sialylation (DIB Fig. 2B). As expected, negligible or no bands were observed when immunopurified MUC1 and EGFR were first sialidase-treated and then precipitated with MAL-I (DIB Fig. 2B). The increased sialylation of MUC1 and EGFR was a least in part due to α2→3 sialic acids because these immunoprecipitated glycoproteins were further precipitated by MAL-I. To confirm MAL-I specificity towards Neu5Acα2→3Gal, equal amounts of desialylated immunoprecipitated glycoproteins (asialoMUC1 and asialoEGFR) were precipitated with MAL-I and subjected to Western blot followed by immunodetection (DIB Fig. 2B).

## 2 Experimental design, materials and methods

### 2.1 Materials

Sialic acid (N-acetyl-5-neuraminic acid, Neu5Ac) was obtained from Santa Cruz Biotechnology (USA). Ribonuclease A, insulin, and hydrocortisone were purchased from Sigma-Aldrich (USA). RPMI1640 medium (ATCC modification), HEPES buffer, fetal bovine serum (FBS), and TO-PRO-3 were purchased from Life Technologies Corporation (USA). Phosphate-buffered saline (PBS, 137 mM sodium chloride, 2.7 mM potassium chloride, 4.3 mM disodium phosphate, 1.4 mM monopotassium phosphate, pH 7.5) was obtained from Technova (USA). FITC conjugates of *Maackia amurensis* agglutinin I (MAL-I, specific for Neu5Ac-α2,3Gal) [3], *Sambucus nigra* agglutinin (SNA, specific for Neu5Ac-α2,6Gal), *Triticum vulgaris* agglutinin (WGA, which recognizes Neu5Ac and GlcNAc) were obtained from Vector Laboratories (USA). All other chemicals were purchased from Sigma-Aldrich in analytical grade quality.

### 2.2 Cell lines and culture conditions

Human normal mammary epithelial cell lines MCF10A and HB4A and breast cancer cell lines T47D, MCF7 and MDA MB231 (American Type Culture Collection, USA) were cultured in 175 cm^2^ flasks in RPMI1640 medium (without added antibiotics to avoid sialyltransferase inhibition [1], [4]), supplemented with 1% FBS (the low level of FBS was used to minimize interference from sialic acid scavenged from serum components such as BSA) at 37 °C under 5% CO_2_. For normal cells, the medium also contained 10 µg/mL insulin and 5 µg/mL hydrocortisone. For all experiments, MCF10A, HB4A, T47D, MCF7 and MDA MB231 cells were used within the first three passages, incubated 72 h to reach mid-exponential growth phase, and harvested by treatment with 5 mL of buffer containing 0.54 mM EDTA, 154 mM NaCl, and 10 mM N-2-hydroxyethylpiperazine-N-2-ethane sulfonic acid (HEPES), pH 7.4 for <5 min at 37 °C.

### 2.3 Nutrient deprivation and Neu5Ac treatment

*Protocol for nutrient deprivation of cells in suspension:* Step 1, cell cultures were harvested as described above. Step 2, cells were resuspended in serum-free RPMI-1640 medium and pelleted by centrifugation at 900*g* for 5 min; during this step cells experience 10 min of nutrient deprivation in serum-free media without Neu5Ac. Step 3, the cells were rinsed twice in 37 °C PBS by centrifugation for 5 min and 1 mL aliquots of 1×10^4^ cells were pipetted gently into 15 mL BD Falcon tubes; during this step the cells experience an additional 20 min of nutrient-deprivation. Step 3, cell suspension aliquots corresponding to 10^4^ cells mL^−1^ were equilibrated in tubes containing Neu5Ac-PBS buffer by placing the tubes with opened caps for 60 min in a humidified incubator at 37 °C and 5% CO_2_ with continuous shaking at 30 strokes per minute; during this Neu5Ac-supplementation step, negative controls were maintained in non-supplemented PBS. Step 4, the Neu5Ac-PBS solution was decanted, the tubes were gently tapped to loosen the gravity-pelleted cells, and then rinsed twice in warm (37 °C) PBS followed by pelleting by 5 min of centrifugation each time; this process provided an additional 30 min of nutrient-deprivation in the absence of supplemental Neu5Ac. The entire 5 step process results in 2 h of nutrient deprivation, after which the cells were analyzed by the methods listed below with the exception of the lectin staining which used cells that were nutrient-deprived under adherent conditions. *Protocol for nutrient deprivation of adherent cells*: Step 1, cells were cultured on sterile glass microscope cover slips for two days. Step 2, the cover slips were placed in a sterile plastic rack in warm (37 °C) PBS buffer for 30 min, then the PBS was replaced with Neu5Ac-PBS solution for 60 min to provide Neu5Ac supplementation (controls were maintained in non-supplemented PBS), and then the cell-laden cover slips were placed back in PBS buffer for 30 min. All incubations were performed in a humidified incubator at 37 °C and 5% CO_2_ with continuous shaking at 30 strokes per minute. Overall this process mimics the non-adherent treatment conditions with respect to the duration of nutrient-deprivation (2 h total) and Neu5Ac supplementation (60 min). In addition to the nutrient-deprivation protocols, “nutrient-happy” control experiments were performed where the cells were maintained in serum containing medium and treated with Neu5Ac (or not for the Neu5Ac(-) controls) as described above.

### 2.4 Flow cytometry analysis

For this purpose, cells were fixed by suspending them in 70% (*v/v*) ethanol and stored at 4 °C for 15 min, washed twice with cold PBS, and then placed in 96-well plates (1×10^4^ cells per well) [1]. The cells were then stained with the fluorescein isothiocyanate-labeled lectins (SWGA, WGA, SNA and MAL-I). For the comparison of mean fluorescence intensities, the instrument settings for fluorescence and compensation were the same for all experiments. Data were collected from at least 10,000 cells for each sample.

### 2.5 Cell viability assay

Cells were harvested as described above without fixation. Cell pellets were resuspended in PBS supplemented with 1 mg/mL propidium iodide (PI) and incubated for 5 min at ambient temperature [1], [2]. Cells were analyzed by flow cytometry on BD Accuri C6 flow cytometer with CFlow Plus operating software (BD Biosciences, USA). The proportion of dead and living cells was determined as the percentage of PI-stained cells. The MTT assay was used to measure changes in cell viability and proliferation for 5 days after returning the Neu5Ac-treated and untreated cells to the complete medium. The formazan dye produced after DMSO solubilization was quantified at 560 nm using a multiwell scanning spectrophotometer (Bio-Rad, USA).

### 2.6 Lectin staining and cell imaging

Cells were grown on the surface of a cover slip and the adherent cells were fixed with 70% ice-cold ethanol for 15 min [1], [2]. After washing with PBS, cells were stained with different FITC-labeled lectins (5 µg/mL) for 1 h. After staining, cells were further treated with Ribonuclease A (10 µg/mL) and the nuclei were counter stained with TO-PRO-3. Images were captured on DV elite imaging system and merged using softWoRx DMS from Applied Precision (Applied Precision, USA).

### 2.7 Immuno-precipitation and lectin-precipitation followed by Western blotting

Cells were lysed in Triton X-100 lysis buffer (10 mM Tris–HCl [pH 8.0], 5 mM ethylenediaminetetraacetic acid, 320 mM sucrose, 1% Triton X-100, 1 mM PMSF, 2 mM DTT, 1 µg/mL leupeptin, 1 µg/mL aprotinin) and then incubated on ice for 15 min [1]. Following centrifugation, the supernatant was collected and protein concentrations were determined by BCA protein assay kit (Pierce). For immuno-precipitation, each cell extract (100 µg of total protein) was incubated with 1 µg of either anti-MUC1 antibody [Mucin1 (sc-7313 Santa Cruz)] or anti-EGFR antibody [epidermal growth factor receptor (EGFR 2232 Cell Signaling)]. The precipitated protein was subjected to SDS-PAGE and Western blotting onto PVDF membranes (Pierce). The blots were then probed with the corresponding primary antibodies followed by secondary antibody-horseradish peroxidase (HRP) conjugate (Santa Cruz) and signals were detected by ECL system (Pierce). Negative control includes PBS instead of primary antibody. For β-Actin control, anti-β-Actin antibody from Santa Cruz (sc-47778) was used. For precipitation of α2→3-sialylated glycoproteins with MAL-I, equal amount of immunoprecipitated MUC1 and EGFR was incubated with 1 μg lectin. The precipitated protein was separated on SDS-PAGE followed by Western blot detection with anti-MUC1 and anti-EGFR antibodies as described above. Immuno- or lectin-precipitation experiments were also performed with the desialylated crude protein extract (for immunoprecipitation) or immunopurified MUC1 and EGFR (for MAL-I precipitation) and similar blots were prepared. For desialylation, the cell extract was incubated with neuraminidase (100 mU/mL) for 1 h at 37 °C.

## Competing financial interests

GlycoMantra, Inc. filed a provisional patent entitled “Exploiting nutrient deprivation for modulation of glycosylation – research, diagnostic, therapeutic, and biotechnology applications” in August, 2015. H.A., H.A.B., and K.J.Y. were listed as inventors. There are no other competing financial interests.
